# Kinetic modeling of anaerobic degradation of plant-derived aromatic mixtures by *Rhodopseudomonas palustris*

**DOI:** 10.1007/s10532-021-09932-3

**Published:** 2021-03-06

**Authors:** Yanjun Ma, Timothy J. Donohue, Daniel R. Noguera

**Affiliations:** 1 Great Lakes Bioenergy Research Center, Wisconsin Energy Institute, University of Wisconsin-Madison, Madison, WI 53726 USA; 2 Department of Bacteriology, University of Wisconsin-Madison, Madison, WI 53706 USA; 3 Department of Civil and Environmental Engineering, University of Wisconsin-Madison, Madison, WI 53706 USA

**Keywords:** Kinetic modeling, *Rhodopseudomonas palustris*, Plant-derived aromatics, Anaerobic degradation, Co-metabolism, Substrate inhibition

## Abstract

*Rhodopseudomonas palustris* is a model microorganism for studying the anaerobic metabolism of aromatic compounds. While it is well documented which aromatics can serve as sole organic carbon sources, co-metabolism of other aromatics is poorly understood. This study used kinetic modeling to analyze the simultaneous degradation of aromatic compounds present in corn stover hydrolysates and model the co-metabolism of aromatics not known to support growth of *R. palustris* as sole organic substrates. The simulation predicted that *p*-coumaroyl amide and feruloyl amide were hydrolyzed to *p*-coumaric acid and ferulic acid, respectively, and further transformed via *p*-coumaroyl-CoA and feruloyl-CoA. The modeling also suggested that metabolism of *p*-hydroxyphenyl aromatics was slowed by substrate inhibition, whereas the transformation of guaiacyl aromatics was inhibited by their *p*-hydroxyphenyl counterparts. It also predicted that substrate channeling may occur during degradation of *p*-coumaroyl-CoA and feruloyl-CoA, resulting in no detectable accumulation of *p*-hydroxybenzaldehyde and vanillin, during the transformation of these CoA ligated compounds to *p-*hydroxybenzoic acid and vanillic acid, respectively. While the simulation correctly represented the known transformation of *p*-hydroxybenzoic acid via the benzoyl-CoA pathway, it also suggested co-metabolism of vanillic acid and syringic acid, which are known not to serve as photoheterotrophic growth substrate for *R. palustris*.

**Supplementary Information:**

The online version of this article (10.1007/s10532-021-09932-3) contains supplementary material, which is available to authorized users.

## Introduction

As the largest renewable feedstock comprising aromatics, lignin in lignocellulosic biomass is increasingly recognized as a great potential source of valuable industrial and commercial products (Ragauskas et al. 2014; Tuck et al. 2012). The structure of lignin is derived from three monomers differing in their degree of methoxylation (*p*-coumaroyl, coniferyl, and sinapyl alcohols), which result in the corresponding *p*-hydrophenyl (H), guaiacyl (G), and syringyl (S) aromatic units in lignin (Davis et al. 2016; Wong 2009). Depolymerization of lignin generates highly heterogeneous mixtures of aromatic monomers, which makes it challenging for extracting and recovering individual products. Microbial treatment has been considered as a possible strategy to covert the heterogeneous mixtures of plant-derived aromatic to a few or even a single compound that can be recovered (Gall et al. 2017; Perez et al. 2019). We have a general interest in identifying microbial routes to transform plant-derived aromatic compounds by microorganisms that could be engineered to make single products from heterogeneous mixtures. The purple non-sulfur bacterium *Rhodopseudomonas palustris* is one of such model microorganisms, which is known to be metabolically versatile in utilizing aromatic compounds under anaerobic conditions (Austin et al. 2015).

The anaerobic metabolism of aromatic compounds by *R. palustris* has been extensively studied and demonstrated to occur via the benzoyl-CoA pathway (Egland et al. 1997; Gall et al. 2013; Harwood et al. 1998). In this pathway, one of several aromatic acids are activated by ligation of Coenzyme A (CoA), and then, the aromatic ring undergoes sequential reduction until cleavage, leading to further transformation to acetyl-CoA, which enters central metabolism. Benzoic and *p*-hydroxybenzoic acids can sustain anaerobic photoheterotrophic growth of *R. palustris* when provided as sole organic carbon sources. Other aromatic compounds known to support anaerobic *R. palustris* growth as sole organic substrates are initially transformed to benzoic acid, *p*-hydroxybenzoic acid, or to their CoA ligated forms. For instance, *p*-coumaric acid has been shown to be metabolized via the benzoyl-CoA pathway after enzymes in upper pathways convert *p*-coumaric to *p*-hydroxybenzoic acid (Hirakawa et al. 2012; Pan et al. 2008). In addition, cinnamic acid, 3-phenylpropionic acid, and 5-phenylvaleric acid have been shown to undergo ß-oxidation to benzoyl-CoA before entering the benzoyl-CoA pathway (Elder et al. 1992).

The existing literature on anaerobic degradation of aromatics by *R, palustris* shows that aromatic acids with substitutions other than a hydroxyl at the *para* position to the carboxylic group cannot serve as sole organic carbon sources for photoheterotrophic growth of *R. palustris* (Harwood and Gibson 1988). There is a recent report of an *R. palustris* strain evolved to degrade syringic acid, which does not use the benzoyl-CoA pathway (Oshlag et al. 2020). In addition, even though there is a limited number of aromatic compounds that support photoheterotrophic growth as sole organic carbon sources, *R. palustris* can metabolize a broad set of aromatic compounds when multiple aromatics are present (Austin et al. 2015; Gall et al. 2013). For instance, while it cannot be used as a sole organic carbon source, protocatechuic acid (3,4-dihydroxybenzoic acid) has been shown to be degraded via the benzoyl-CoA pathway if either benzoic acid or *p*-hydroxybenzoic acid is provided as a co-substrate (Gall et al. 2013). Furthermore, the ability of *R. palustris* to co-metabolize substrates that cannot serve as sole organic substrates for photoheterotrophic growth appears to be a feature not limited to aromatic compounds (Govindaraju et al. 2019).

We have previously reported on the degradation of multiple aromatic compounds when *R. palustris* is grown on aromatic-containing hydrolysates produced from lignocellulosic biomass (Austin et al. 2015). The most abundant aromatic compounds in these hydrolysates were *p*-coumaroyl amide and feruloyl amide, with 16 other aromatic compounds detected at lower concentrations (Austin et al. 2015). In these experiments, *R. palustris* utilized most of the aromatics in the hydrolysates with extracellular accumulation of *p*-hydroxybenzoic acid, vanillic acid, and protocatechuic acid. Since the ability of *R. palustris* to anaerobically degrade most of the aromatic compounds found in these hydrolysates has not been previously documented, either as sole substrates or co-substrates, we used kinetic modeling to investigate *R. palustris* metabolism of these compounds in the batch experiments reported by Austin et al. (2015). The goal was to generate hypotheses of degradation pathways and factors that influence co-metabolism of aromatic compounds by *R. palustris*. These kinetic models propose pathways that are consistent with existing knowledge and best fit the trends of aromatic metabolism observed when this bacterium grows in the presence of these biomass hydrolysates.

## Materials and methods

### Batch reactor

As described in Austin et al. (2015), the batch reactor contained 1000 mL of ammonia fiber expansion (AFEX) treated corn stover hydrolysates (ACSH) and was inoculated with 20 mL *R. palustris* CGA009 pregrown in minimal medium. The reactor was operated anaerobically for about 192 h at 28 °C and exposed to continuous light. Data for the batch experiment was obtained from Austin et al. (2015), including cell optical density measured at 600 nm (OD_600_), concentration of aromatic compounds in the starting ACSH (Table 1 and Fig. S1) and over time.

**Table 1:** Concentration of aromatic compounds identified in the ammonia fiber expansion (AFEX) treated corn stover hydrolysates (ACSH) used by Austin et al. (2015)

*p*-Hydroxyphenyl		Guaiacyl		Syringyl		Other	
Compound	Concentration (µmol l^−1^)	Compound	Concentration (µmol l^−1^)	Compound	Concentration (µmol l^−1^)	Compound	Concentration (µmol l^−1^)
*p*-Coumaroyl amide	2090	Feruloyl amide	1040	Sinapoyl amide	Not detected	Benzoic acid	160
*p*-Coumaric acid	446	Ferulic acid	20.9	Sinapic acid	Not detected	Protocatechuic acid	6.0
*p*-Hydroxybenzaldehyde	59.4	Vanillin	43.3	Syringaldehyde	5.8		
*p*-Hydroxybenzamide	20.6	Vanillamide	60.5	Syringamide	32.6		
*p*-Hydroxyacetophenone	1.8	Acetovanillone	8.2	Acetosyringone	5.4		
*p*-Hydroxybenzoic acid	47.8	Vanillic acid	27.0	Syringic acid	6.9		

Chemical structures of the compounds are shown in Fig. S1

### Kinetic models

The rate of change of the concentration of each aromatic compound in the batch reactor is expressed as:

1 $$\frac{dS}{dt}={r}_{p}X-{r}_{s}X$$

where S stands for substrate concentration (μmol l^−1^), X represents biomass concentration (mg l^−1^), and r_p_ and r_s_ stand for specific substrate production and consumption rates (μmol mg^−1^ h^−1^), respectively. Biomass concentrations were estimated from measured OD_600_ using a correlation determined in separate experiments. The Gompertz function (Gompertz 1825) was used to model X in the batch reactor as a function of time (Fig. S8).

Substrate production and consumption rates were simulated using either first order rates (Eq. 2) or rates that account for inhibition by a secondary substrate (Eq. 3). In these equations, *k* is the first order reaction rate (l mg^−1^ h^−1^), k_i_ is the inhibition factor (μmol l^−1^), S_i_ is the concentration of inhibitory substrate (μmol l^−1^), and all other terms are as defined above.

2 $$r=kS$$

3 $$r=k\left(\frac{{k}_{i}}{{k}_{i}+{S}_{i}}\right)S$$

The differential equations representing substrate concentration over time (Eq. 1) were solved numerically using Euler’s method (Atkinson 1989) with a time step of 1 h, and expressed as a user-defined function in R (R Core Team 2019). The best-fit parameters k and k_i_ were estimated by minimizing the Residual Sum of Squares (RSS) between the modeled and measured substrate concentrations using the optim function in R (R Core Team 2019).

## Results and discussion

### Proposed pathways for anaerobic co-metabolism of p-hydroxyphenyl, guaiacyl and syringyl type aromatics by *R. palustris*

The abundant aromatic compounds identified in the lignocellulosic biomass hydrolysates used by Austin et al. (2015) included *p*-hydroxyphenyl (H; no methoxy substitution), guaiacyl (G; with one *meta*-methoxy substitution) and syringyl (S; with two *meta*-methoxy substitutions) type aromatics (Table 1, Fig. S1) derived from pretreatment of corn stover using the ammonia fiber expansion (AFEX) pretreatment process (Teymouri et al. 2004). Among these compounds, it is well established that benzoic acid, *p*-hydroxybenzoic acid, *p*-coumaric acid, and protocatechuic acid are metabolized by *R. palustris* anaerobically through the benzoyl-CoA pathway (Egland et al. 1997; Gall et al. 2013; Harwood et al. 1998; Hirakawa et al. 2012; Pan et al. 2008). In contrast, pathways for the degradation of the most abundant aromatic compounds in the hydrolysate, *p*-coumaroyl amide and feruloyl amide, have not been elucidated.

**Fig. 1:**
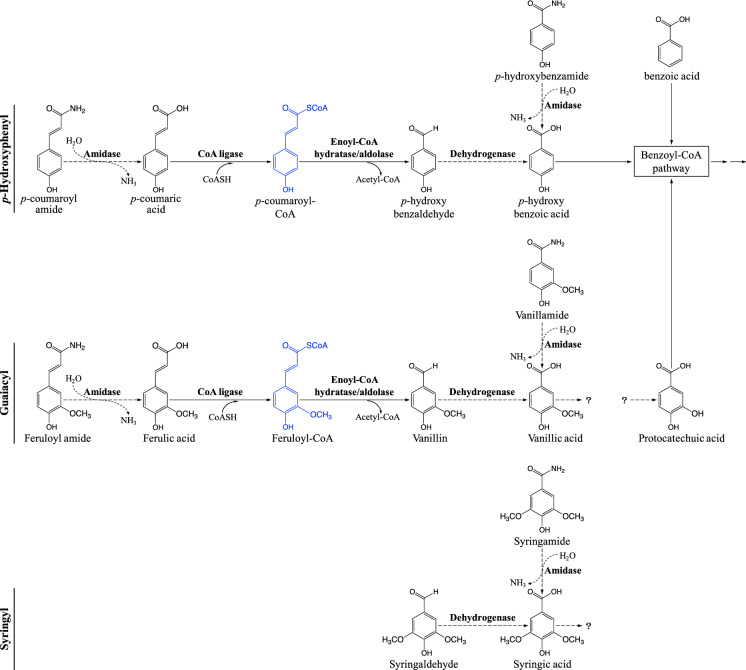
Proposed degradation pathways for plant-derived aromatic compounds detected in the experiment of Austin et al. (2015). Solid arrows indicated reactions that are experimentally demonstrated in *R. palustris*. Dashed arrows indicated hypothetical reactions of this study. Compounds colored in blue (*p*-coumaroyl-CoA and feruloyl-CoA) represent intermediates not measured

To inform our modeling of aromatic co-metabolism, we propose here tentative pathways for *p*-coumaroyl amide and feruloyl amide degradation (Fig. 1) based on existing evidence and hypothetical reactions. Since these aromatic amides were completely transformed by *R. palustris* (Austin et al. 2015) and it is known that bacterial amidases can hydrolyze a broad range of amides (including aromatic amides) to produce the corresponding acids (Hirrlinger et al. 1996; Ismailsab et al. 2017; Ruan et al. 2016), we propose that *p*-coumaroyl amide and feruloyl amide are hydrolyzed by amidases to *p*-coumaric acid and ferulic acid, respectively (Fig. 1). Once deamidated, we propose that these aromatic acids can be subsequently activated by CoA ligation. Then, with an enoyl-CoA hydratase/aldolase, *p*-coumaroyl-CoA and feruloyl-CoA can be converted to the respective aldehydes *p*-hydroxybenzaldehyde and vanillin. Pan et al. (2008) described the CoA ligase (CouB) and enoyl-CoA hydratase/aldolase (CouA) gene products that convert *p*-coumaric acid to *p*-hydroxybenzaldehyde. Activity of these enzymes was also demonstrated with ferulic acid to yield vanillin (Hirakawa et al. 2012), and therefore, we hypothesize that in mixtures containing coumaric acid and ferulic acid, CuoAB can transform both acids to their corresponding aldehydes (Fig. 1).

Since the degradation of *p*-coumaric acid by *R. palustris* has been demonstrated to occur via the benzoyl-CoA pathway (Pan et al. 2008), we propose that *p*-hydroxybenzaldehyde is further transformed to *p*-hydroxybenzoic acid by an aldehyde dehydrogenase, in agreement to the pathway suggested by Pan et al. (2008). Then, *p*-hydroxybenzoic acid is activated by CoA ligation and enters the benzoyl-CoA pathway according to well established pathways (Egland et al. 1997; Harwood et al. 1998). On the other hand, ferulic acid has been shown to support photoheterotrophic growth of *R. palustris* even though this aromatic acid is only partially transformed to vanillic acid; the alkyl chain serves as the organic carbon source for growth (Harwood and Gibson 1988). Therefore, there is no evidence to suggest a potential pathway for the complete degradation of ferulic acid by *R. palustris*. Instead, we propose that ferulic acid is converted to vanillic acid via vanillin by CuoAB (Hirakawa et al. 2012) and an aldehyde dehydrogenase (Pan et al. 2008). In the experiments of Austin et al. (2015), vanillic acid accumulated as an extracellular product, but it is not known whether its accumulation represents the complete inability of *R. palustris* to degrade vanillic acid or a slow and incomplete degradation of this product. Experiments with vanillic acid as the sole carbon source show that this substrate does not support anaerobic growth of *R. palustris* (Harwood and Gibson 1988; Oshlag et al. 2020), and thus, if vanillic acid was degraded, we hypothesize that it occurred by co-metabolism.

The hydrolysate used in the Austin et al. (2015) experiments also contained lower concentrations of the aromatic amides *p*-hydroxybenzamide, vanillamide, and syringamide. These aromatic substrates were partially degraded by *R. palustris* (Austin et al. 2015); thus, we hypothesize that they can be hydrolyzed by amidases to *p*-hydroxybenzoic acid, vanillic acid, and syringic acid, respectively (Fig. 1). Furthermore, by analogy to the transformation of *p*-hydroxybenzaldehyde and vanillin, we hypothesize that syringaldehyde is converted to syringic acid (Fig. 1), which accumulated extracellularly in the experiments of Austin et al. (2015). Syringic acid is not a substrate that supports anaerobic growth of wild type *R. palustris*. However, we recently adapted an *R. palustris* strain able to photoheterotrophically grow on this substrate as the sole organic carbon source (Oshlag et al. 2020). This adaptation took several rounds of selection that were not used in the experiments of Austin et al. (2015), in which syringic acid degradation was not apparent, and therefore, we hypothesize that syringic acid was not significantly co-metabolized in the experiments of Austin et al. (2015).

Three additional aromatic compounds were detected in the hydrolysates used by Austin et al. (2015), *p*-hydroxyacetophenone, acetovanillone, and acetosyringone. Of these compounds, the extracellular concentration of *p*-hydroxyacetophenone increased during growth in hydrolysates, whereas the concentrations of acetovanillone and acetosyringone remained constant throughout the study (Fig. S2). Given that these compounds were found at low concentrations and that there is no evidence for their degradation by *R. palustris*, we did not include these substrates in the kinetic analysis described below.

### Kinetic simulation of the metabolism of p-hydroxyphenyl and guaiacyl type aromatics

**Fig. 2:**
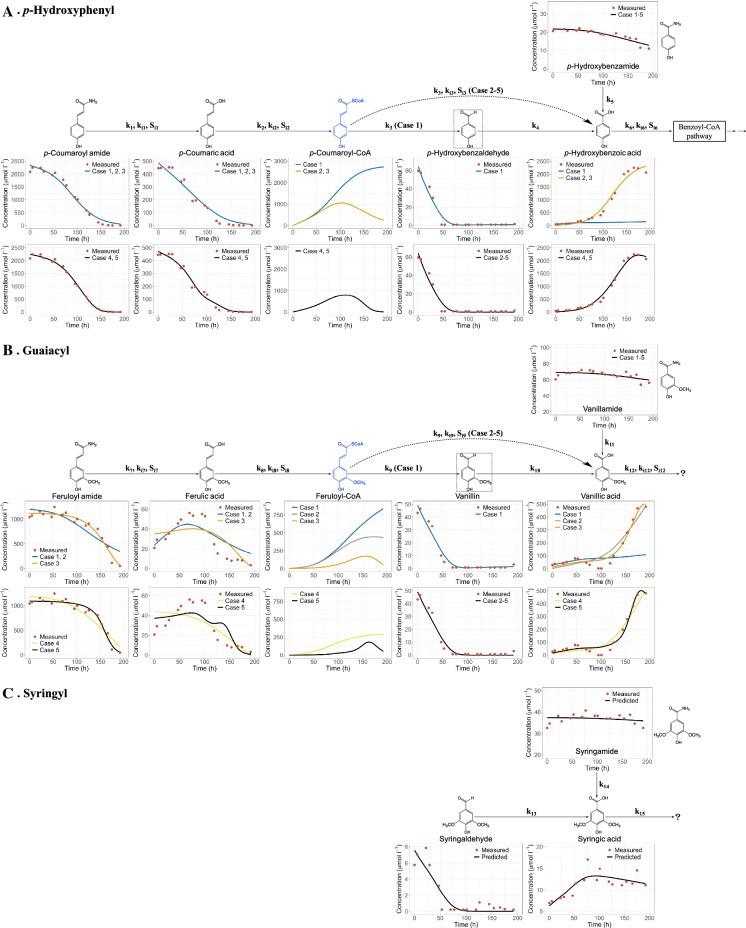
Kinetic modeling of **a** *p*-hydroxyphenyl, **b** guaiacyl, and **c** syringyl aromatics with alternative models exploring factors that may influence their transformation rates. For *p*-hydroxyphenyl and guaiacyl aromatics, Case 1 and 2: no inhibition effects; Case 3: H type aromatics inhibit degradation of structurally similar G type aromatics; Case 4: substrate inhibition of both H and G type aromatics; Case 5: substrate inhibition of H type aromatics, and H type aromatics inhibit degradation of structurally similar G type aromatics. Case 1: no substrate channeling; Case 2–5: substrate channeling in degradation of *p*-coumaroyl-CoA and feruloyl-CoA. Degradation rate (k), inhibition factor (k_i_) and inhibitory substrate (S_i_) of each reaction are shown in Table S1

We used the time series results presented in Austin et al. (2015) to evaluate whether the transformation pathways proposed in Fig. 1 were appropriate for simulating the simultaneous degradation of aromatic compounds by *R. palustris* and to elucidate factors that may influence their transformation rates. We first simulated each reaction step with a simple first order rate equation (see Materials and Methods), and then evaluated the impact of other assumptions on the goodness of fit between experimental results and model outputs (Table 2). Model outputs and optimized parameters are collectively shown in Fig. 2 and Table S1, and separately shown in the supplementary document (Fig. S3–S7). An assumption in these models is that the observed extracellular concentration of pathway intermediates provides a reasonable representation of the balance between the rates of production and degradation of each intermediate. This assumption was used for all intermediates that were extracellularly measurable. However, there was no measured concentration for the CoA-ligated intermediates, which only accumulate intracellularly, and therefore, the models assumed a hypothetical accumulation of these compounds.

**Table 2:** Goodness of fit between experimental results and model outputs expressed as Residual sum of squares (RSS) in simulating metabolism of *p*-hydroxyphenyl (H) and guaiacyl (G) type aromatics

Simulation*	*p*-Hydroxyphenyl (H)					Guaiacyl (G)					Total
	*p*-Coumaroyl amide	*p*-Coumaric acid	*p*-Hydroxy benzaldehyde	*p*-Hydroxy benzamide	*p*-Hydroxy benzoic acid	Feruloyl amide	Ferulic acid	Vanillin	Vanillamide	Vanillic acid	
Case1	242,976	22,457	233	22	22,334,583	372,602	1967	157	246	420,174	23,395,418
Case2	242,976	22,457	239	22	297,626	372,602	1967	167	246	44,864	983,166
Case3	242,976	22,457	239	22	297,626	89,068	2308	167	246	17,218	672,327
Case4	60,701	10,982	239	22	157,352	199,037	2670	167	246	27,876	459,293
Case5	60,701	10,982	239	22	157,352	83,872	2193	167	246	14,650	330,425

*Case 1 and 2: no inhibition effects, Case 3: H type aromatics inhibit degradation of structurally similar G type aromatics, Case 4: substrate inhibition of both H and G type aromatics, Case 5: substrate inhibition of H type aromatics, and H type aromatics inhibit degradation of structurally similar G type aromatics. Case 1: no substrate channeling, Case 2–5: substrate channeling in degradation of *p*-coumaroyl-CoA and feruloyl-CoA

The initial model, which used first order rates for all reactions (Case 1), resulted in good fits between predictions and most of the measured extracellular aromatics, but failed to predict the observed accumulation of *p*-hydroxybenzoic and vanillic acids (Fig. 2, Fig. S3). The fit of the model to the measured ferulic acid concentrations was deficient, likely because the concentrations of ferulic acid were much lower than the concentrations of other aromatics, and in general, the best-fitting technique will place less weight on matching simulated and experimental concentrations for this aromatic compound. The obtained best-fit reaction rates with this model (Table S1) indicated that the transformation rate for the CoA ligated intermediates was 2 to 3 orders of magnitude lower than the reaction rates of the upstream (*p*-coumaric acid and ferulic acid) and the downstream (*p*-hydroxybenzaldehyde and vanillin) intermediates. Therefore, this simulation predicted a bottleneck in the pathways, with accumulation of *p*-coumaroyl-CoA and feruloyl-CoA, and since the model was not able to predict the accumulation of *p*-hydroxybenzoic and vanillic acids (Fig. 2), we interpreted these results as being an incorrect hypothetical accumulation of the CoA-ligated intermediates.

To improve the accuracy of this prediction, another simulation (Case 2) considered the direct transformation of *p*-coumaroyl-CoA and feruloyl-CoA to *p*-hydroxybenzoic and vanillic acid, respectively, without accumulation of *p*-hydroxybenzaldehyde or vanillin (Fig. 2, Fig. S4). This assumption is justified by experimental observations of little extracellular accumulation of vanillin during the transformation of ferulic acid to vanillic acid in other organisms, presumably due to rapid metabolism or substrate channeling to prevent the toxicity of the intermediate aldehyde (Fitzgerald et al. 2004; Priefert et al. 2001). Substrate channeling has been reported to occur in other microbial transformations in which the accumulation of toxic intermediates is minimized or prevented (Abernathy et al. 2017). With this modification, the model output (Fig. 2) accurately predicts the observed extracellular accumulation of *p*-hydroxybenzoic and vanillic acids. Compared to the simulation of Case 1, the revised model predicts accumulation and degradation of the CoA ligated intermediates and rate constants for these steps that are of similar order of magnitude as other reactions in the pathway (Table S1). Because of the improvement observed in Case 2, substrate channeling in degradation of *p*-coumaroyl-CoA and feruloyl-CoA was assumed in all subsequent refinements of the model (Fig. 2, Fig. S5–S7).

By comparing Case 2 simulation results with the experimental observations of G type aromatics metabolism, we noted an apparent lag in the experimental degradation of feruloyl amide that was not captured by the model (Fig. 2). Since there is experimental evidence that the CuoAB enzymes, responsible for the transformations from *p*-coumaric acid to *p*-hydroxybenzaldehyde, are active on the transformation of ferulic acid to vanillin (Hirakawa et al. 2012), we simulated a situation in which the metabolism of the G type aromatics is inhibited by the binding of these H type aromatics to these enzymes. That is, it may be possible that G aromatics can bind to these H-pathway enzymes at a lower affinity due to the presence of the *meta*-methoxy substitution on the ring. Therefore, we modified the kinetic expressions in the modeling of the degradation of feruloyl amide, ferulic acid and feruloyl-CoA by simulating competitive inhibition (See Materials and Methods) by *p*-coumaroyl amide, *p*-coumaric acid, and *p*-coumaroyl-CoA, respectively (Case 3; Fig. 2, Fig. S5).

By allowing competitive inhibition of G type aromatics by the corresponding H type aromatics, the simulation in Case 3 greatly improved the fit of experiments to simulations, specifically for feruloyl amide and vanillic acid (Table 2). The accumulation of vanillic acid in the Case 2 simulation (Fig. 2) was accompanied by a prediction that this aromatic was not degraded (i.e., the degradation rate was zero; Table S1). This is consistent with previous reports that this aromatic cannot be metabolized by wild type *R. palustris* anaerobically (Harwood and Gibson 1988; Oshlag et al. 2020). However, in the Case 3 simulation, the best-fit solution required vanillic acid to have a non-zero degradation rate (Table S1), suggesting co-metabolism of vanillic acid in the aromatic mixture. Since a pathway for vanillic acid degradation cannot be inferred from the available data, we used the simple first order rate to describe vanillic acid degradation instead of assuming competitive inhibition by *p*-hydroxybenzoic acid. The same description was also applied in modeling vanillic acid degradation in subsequent cases.

While the simulation of Case 3 provided a good fit to the experimental trends, experiments and simulations of *p*-coumaroyl amide and *p*-coumaric acid diverged after 100 h, with experimental observations showing a faster degradation rate than predicted by this version of the model (Fig. 2). To further attempt to improve the match of simulations to experiments, we explored the hypothesis of substrate inhibition. That is, we modified the kinetic expression so that the aromatic compound degradation was inhibited at high substrate concentrations (see Materials and Methods). This modification was implemented for *p*-coumaroyl amide, *p*-coumaric acid, *p*-coumaroyl-CoA, and *p*-hydroxybenzoic acid belonging to H type aromatics, and feruloyl amide, ferulic acid, feruloyl-CoA and vanillic acid for the G type aromatics. This instance of the model (Case 4; Fig. 2, Fig. S6) best fitted the experimental results of each H type aromatics, but the fit to G type aromatics was generally poorer than in Case 3 (Table 2).

By comparing the assumptions in Cases 2–4, a hybrid of kinetic expressions was simulated, in which H type aromatics were dominantly impacted by substrate inhibition (as simulated in Case 4), and G type aromatics were impacted by competitive inhibition (as simulated in Case 3). This instance of the model (Case 5; Fig. 2, Fig S7) best fitted experimental results of both H and G type aromatics compared with previous cases (Table 2).

### Simulation of vanillic acid and benzoic acid metabolism

**Fig. 3:**
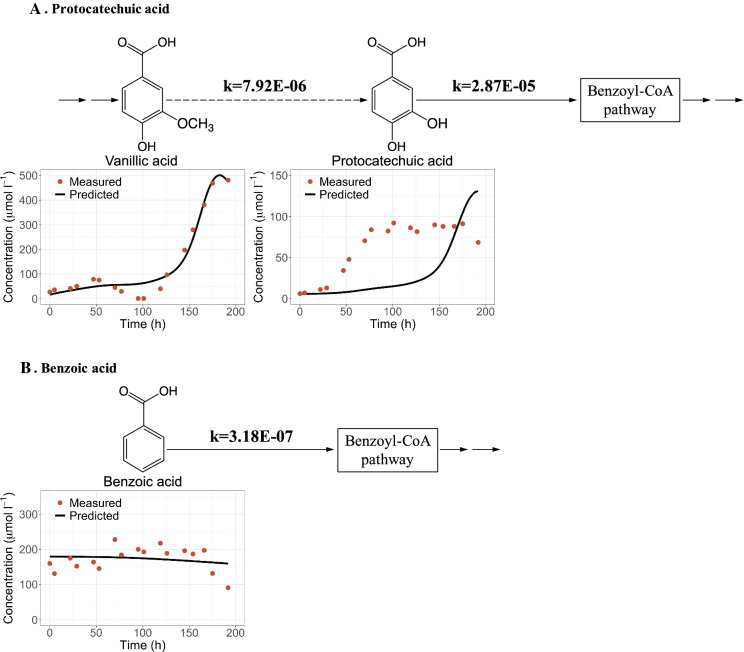
Comparison of experimental and modeling results for **a** protocatechuic acid production from vanillic acid assuming first order rates of protocatechuic production and consumption, and **b** benzoic acid consumption assuming first order degradation rate. The units of the estimated first order rates are l mg^−1^ h^−1^

Overall, our simulations supported the hypothesis that vanillic acid is consumed in the experiments. Although no vanillic acid degradation was simulated by Cases 1 and 2, a positive degradation rate was predicted as we refined the simulations in Cases 3–5 (Table S1). Vanillic acid is known not to be degraded anaerobically by *R. palustris* as sole organic carbon source (Harwood and Gibson 1988; Oshlag et al. 2020). Therefore, we propose that vanillic acid was co-metabolized through a pathway that required the presence of other aromatics, as has been observed previously in this bacterium (Gall et al. 2013). Current knowledge on metabolism of vanillic acid by other bacteria involves demethylation and conversion to protocatechuic acid either by vanillate demethylase (VanA and VanB) which is reported to require oxygen (Chen et al. 2012; Priefert et al. 1997; Segura et al. 1999; Sudtachat et al. 2009; Venturi et al. 1998) or H_4_-folate-dependent aromatic O-demethylases which can be active under anaerobic conditions (Abe et al. 2005; Berman and Frazer 1992; Kaufmann et al. 1998; Naidu and Ragsdale 2001; Nishikawa et al. 1998). We found that simulating the production of protocatechuic acid from vanillic acid did not yield a good fit to the observed extracellular levels of protocatechuic acid (Fig. 3a). Therefore, it is possible that the experimentally observed protocatechuic acid accumulation is not due to vanillic acid degradation, but to the degradation of another aromatic compound in the hydrolysate medium that was not identified.

It is also known that benzoic acid, *p*-hydroxybenzoic acid and protocatechuic acid are all degraded through benzoyl-CoA pathway in *R. palustris* (Egland et al. 1997; Gall et al. 2013; Harwood et al. 1998). The experimental results of Austin et al. (2015) showed extracellular accumulation of both *p*-hydroxybenzoic acid and protocatechuic acid. The benzoic acid concentration remained relatively constant, and a first-order degradation model suggested a relatively low degradation rate (Fig. 3b). The accumulation of *p*-hydroxybenzoic acid (Fig. 2) and the low degradation rate of benzoic acid compared with other H type aromatics (Fig. 3b) indicated that, in the experiments of results of Austin et al. (2015), substrates entering the benzoyl-CoA pathway was a limiting step in the degradation of the aromatic mixtures by *R. palustris*.

### Kinetic simulation of the metabolism of syringyl aromatics

Syringyl type aromatics with longer alkyl chain such as sinapoyl amide or sinapic acid were not detected in these biomass hydrolysates (Table 1, Fig. S1), and therefore, the kinetic modeling of S aromatics was limited to syringamide, syringaldehyde, and syringic acid. A simulation using first order rates for the degradation of these three S aromatics (Fig. 2) showed best fits when there were positive degradation rates for the three metabolites (Table S1). Given the adequate fits observed with the first order rates and the low concentrations of these metabolites, this was the only model used for the S type aromatics. Comparing the transformation rates of the aromatic aldehydes (Table S1), the degradation rates follow the trend of *p*-hydroxybenzaldehyde > vanillin > syringaldehyde. Similarly, the transformation rates of aromatic amides followed the trend of *p*-hydroxybenzamide > vanillamide > syringamide (Table S1). Thus, the kinetic simulations do suggest that a higher number of methoxy substitutions decreases the rate at which these compounds are metabolized. Similar to vanillic acid, syringic acid is known not to be degraded anaerobically by *R. palustris* as sole organic carbon source (Harwood and Gibson 1988). Degradation of syringic acid by other bacteria is usually initiated by demethylation, via demethylase enzymes that typically do not require oxygen as a substrate (Abe et al. 2005; Kasai et al. 2005; Masai et al. 2004; Wu et al. 1988). Using first order degradation rates, our model predicts a small positive rate for syringic acid degradation (Table S1). Although syringyl compounds are found in low concentration in these hydrolysates, this finding can be used to propose that, contrary to our initial hypothesis, syringic acid may be co-metabolized in the aromatic hydrolysate mixture. However, further study is needed to elucidate whether the syringic acid was metabolized via the benzoyl-CoA pathway or other unknown pathways, as suggested by analysis of an evolved strain of *R. palustris* that acquired the ability to use this aromatic acid as a sole carbon source (Oshlag et al. 2020).

### Concluding remarks

The kinetics-based simulation analysis presented here offers predictions for co-metabolism of several aromatic compounds when present in complex mixtures. Although various models have been applied to describe kinetics of co-metabolic degradations (Alvarez-Cohen and Speitel 2001; Wang et al. 2002, 2014), the first order kinetic and rates that account for inhibition by a secondary substrate described in this study provided an applicable method to simulate systems including complex pathways and inhibition effects. Overall, the proposed *p*-hydroxyphenyl, guaiacyl and syringyl degradation pathways (Fig. 1) were supported by the fit between experimental results and simulation outputs. Furthermore, the kinetic analysis suggested that co-metabolism of aromatic compounds may be impacted by substrate inhibition and competitive inhibition, presumably because the ability of *R. palustris* to degrade multiple aromatic compounds is based on having enzymes with broad substrate specificity. Substrate inhibition can impact degradation of H type aromatics, whereas degradation of G type aromatics can be impacted by inhibition from competing H type aromatics. These models make several new predictions for experimental studies to provide a more thorough understanding of the pathways and factors influencing degradation of aromatics by *R. palustris*. Ultimately, the results of future experimental and modeling studies will contribute to furthering the knowledge of aromatic metabolism by *R. palustris* that could enable engineering approaches that improve the ability of this and other bacteria to convert plant-derived aromatic mixtures into valuable products.

## Supplementary Information

Below is the link to the electronic supplementary material.

Supplementary file1 (DOCX 2830 kb)

## Data Availability

The authors confirm that the data supporting the findings of this study are available within the article and its supplementary materials.
