# Supplementary material for: Kinetic modeling of anaerobic degradation of plant-derived aromatic mixtures by Rhodopseudomonas palustris
Source: Biodegradation. 2021 Mar 6;32(2):179–92. doi: 10.1007/s10532-021-09932-3 (PMC7997838; doi:10.1007/s10532-021-09932-3)
Supplement: Supplementary file 1 — Supplementary file1 (DOCX 2830 kb) [file 10532_2021_9932_MOESM1_ESM.docx]

**Supplemental Material for**

**Kinetic modeling of anaerobic degradation of plant-derived aromatic mixtures by *Rhodopseudomonas palustris***

Yanjun Ma^1^, Timothy J. Donohue^1,2^, Daniel R. Noguera^1,3^

^1^ Great Lakes Bioenergy Research Center, Wisconsin Energy Institute, University of Wisconsin, Madison WI 53726

^2^Department of Bacteriology, University of Wisconsin, Madison, WI 53706

^3^Department of Civil & Environmental Engineering, University of Wisconsin, Madison, WI 53706

Corresponding author: Daniel R. Noguera (dnoguera@wisc.edu)

**SUPPLEMENTAL TABLE**

**Table S1.** Best-fit estimates of degradation rates (k) and inhibition factors (k_i_) for *p*-hydroxyphenyl (H), guaiacyl (G) and syringyl (S) aromatics.

| *p*-Hydroxyphenyl (H) | | | | | | | | | | | | | | | | | | | |
| --- | --- | --- | --- | --- | --- | --- | --- | --- | --- | --- | --- | --- | --- | --- | --- | --- | --- | --- | --- |
| Simulation* | *p*-Coumaroyl amide | | |  | *p*-Coumaric acid | | |  | *p*-Coumaroyl-CoA | | |  | *p*-Hydroxy  benzaldehyde |  | *p*-Hydroxy  benzamide |  | *p*-Hydroxybenzoic acid | | |
|  | k_1_ | k_i1_ | S_i1_ |  | k_2_ | k_i2_ | S_i2_ |  | k_3_ | k_i3_ | S_i3_ |  | k_4_ |  | k_5_ |  | k_6_ | k_i6_ | S_i6_ |
| Case1 | 1.00E-05 | - | - |  | 7.70E-05 | - | - |  | 3.49E-08 | - | - |  | 1.03E-04 |  | 1.40E-06 |  | 0 | - | - |
| Case2 | 1.00E-05 | - | - |  | 7.70E-05 | - | - |  | 9.96E-06 | - | - |  | 1.03E-04 |  | 1.40E-06 |  | 5.48E-07 | - | - |
| Case3 | 1.00E-05 | - | - |  | 7.70E-05 | - | - |  | 9.96E-06 | - | - |  | 1.03E-04 |  | 1.40E-06 |  | 5.48E-07 | - | - |
| Case4 | 3.17E-05 | 577 | *p*-Coumaroyl amide |  | 2.39E-04 | 112 | *p*-Coumaric acid |  | 3.78E-05 | 486 | *p*-Coumaroyl-CoA |  | 1.03E-04 |  | 1.40E-06 |  | 1.10E-01 | 0.02 | *p*-Hydroxy  benzoic acid |
| Case5 | 3.17E-05 | 577 | *p*-coumaroyl amide |  | 2.39E-04 | 112 | *p*-Coumaric acid |  | 3.78E-05 | 486 | *p*-Coumaroyl-CoA |  | 1.03E-04 |  | 1.40E-06 |  | 1.10E-01 | 0.02 | *p*-Hydroxy  benzoic acid |
| Guaiacyl (G) | | | | | | | | | | | | | | | | | | | |
|  | Feruloyl amide | | |  | Ferulic acid | | |  | Feruloyl-CoA | | |  | Vanillin |  | Vanillamide |  | Vanillic acid | | |
|  | k_7_ | k_i7_ | S_i7_ |  | k_8_ | k_i8_ | S_i8_ |  | k_9_ | k_i9_ | S_i9_ |  | k_10_ |  | k_11_ |  | k_12_ | k_i12_ | S_i12_ |
| Case1 | 3.33E-06 | - | - |  | 7.97E-05 | - | - |  | 1.30E-07 | - | - |  | 7.15E-05 |  | 3.98E-07 |  | 0 | - | - |
| Case2 | 3.33E-06 | - | - |  | 7.97E-05 | - | - |  | 3.44E-06 | - | - |  | 7.13E-05 |  | 3.98E-07 |  | 0 | - | - |
| Case3 | 5.75E-01 | 0.003 | *p*-Coumaroyl amide |  | 8.32E-00 | 0.0008 | *p*-Coumaric acid |  | 6.76E-01 | 0.02 | *p*-Coumaroyl-CoA |  | 7.13E-05 |  | 3.98E-07 |  | 7.60E-06 | - | - |
| Case4 | 4.04E-01 | 0.01 | Feruloyl amide |  | 1.06E-01 | 0.03 | Ferulic acid |  | 9.58E-06 | * | Feruloyl-CoA |  | 7.13E-05 |  | 3.98E-07 |  | 8.84E-01 | 0.001 | Vanillic acid |
| Case5 | 2.07E-05 | 48 | *p*-Coumaroyl amide |  | 1.61E-03 | 2.08 | *p*-Coumaric acid |  | 3.94E-05 | 1782 | *p*-Coumaroyl-CoA |  | 7.13E-05 |  | 3.98E-07 |  | 7.92E-06 | - | - |
| Syringyl (S) | | | | | | | | | | | | | | | | | | | |
|  |  |  |  |  |  |  |  |  |  |  |  |  | Syring  aldehyde |  | Syringamide |  | Syringic acid |  |  |
|  |  |  |  |  |  |  |  |  |  |  |  |  | k_13_ |  | k_14_ |  | k_15_ |  |  |
| Case1 |  |  |  |  |  |  |  |  |  |  |  |  | 5.48E-05 |  | 1.07E-07 |  | 8.31E-07 |  |  |

^*^ For H and G type aromatics, cases are as defined in footnote of Table 2. For S type aromatics, only first order simulations were performed, corresponding to Case 1 in the other simulations. The symbol “-” indicates that the parameter is not included in the model, and the symbol “*” indicates k_i_>10,000, far larger than corresponding S_i_ and showing that simulation did not predict inhibition. The units of k, k_i_ and S_i_ are l mg^-1^ h^-1^, μmol l^-1^, and μmol l^-1^, respectively.

**SUPPLEMENTAL FIGURES**

**Figure S1.** Chemical structure and concentration of aromatic compounds identified in the ammonia fiber expansion (AFEX) treated corn stover hydrolysates (ACSH) used by Austin et al. (2015).

| ***p*-Hydroxyphenyl** | | | | | |  |
| --- | --- | --- | --- | --- | --- | --- |
|   *p*-coumaroyl  amide  (2090 µmol l^-1^) |   *p*-coumaric  acid  (446 µmol l^-1^) |   *p*-hydroxy-  benzaldehyde  (59.4 µmol l^-1^) |   *p*-hydroxy-  benzamide  (20.6 µmol l^-1^) |   *p*-hydroxy-  acetophenone  (1.8 µmol l^-1^) |   *p*-hydroxy-  benzoic acid  (47.8 µmol l^-1^) | |
| **Guaiacyl** | | | | | |  |
|   Feruloyl amide  (1040 µmol l^-1^) |   Ferulic acid  (20.9 µmol l^-1^) |   Vanillin  (43.3 µmol l^-1^) |   Vanillamide  (60.5 µmol l^-1^) |   Acetovanillone  (8.2 µmol l^-1^) |   Vanillic acid  (27.0 µmol l^-1^) | |
| **Syringyl** | | | | | |  |
|   Sinapoyl amide  (Not detected) |   Sinapic acid  (Not detected) |   Syringaldehyde  (5.8 µmol l^-1^) |   Syringamide  (32.6 µmol l^-1^) |   Acetosyringone  (5.4 µmol l^-1^) |   Syringic acid  (6.9 µmol l^-1^) | |
| **Others** | | | | | |  |
|   Benzoic acid  (160 µmol l^-1^) |   Protocatechuic acid  (6.0 µmol l^-1^) |  |  |  |  | |

**Figure S2.** Experimental results from Austin et al. (2015) for *p*-hydroxyacetophenone, acetovanillone and acetosyringone. *p*-Hydroxyacetophenone slightly increased but the source is unknown. Little consumption was seen for acetovanillone and acetosyringone.

**
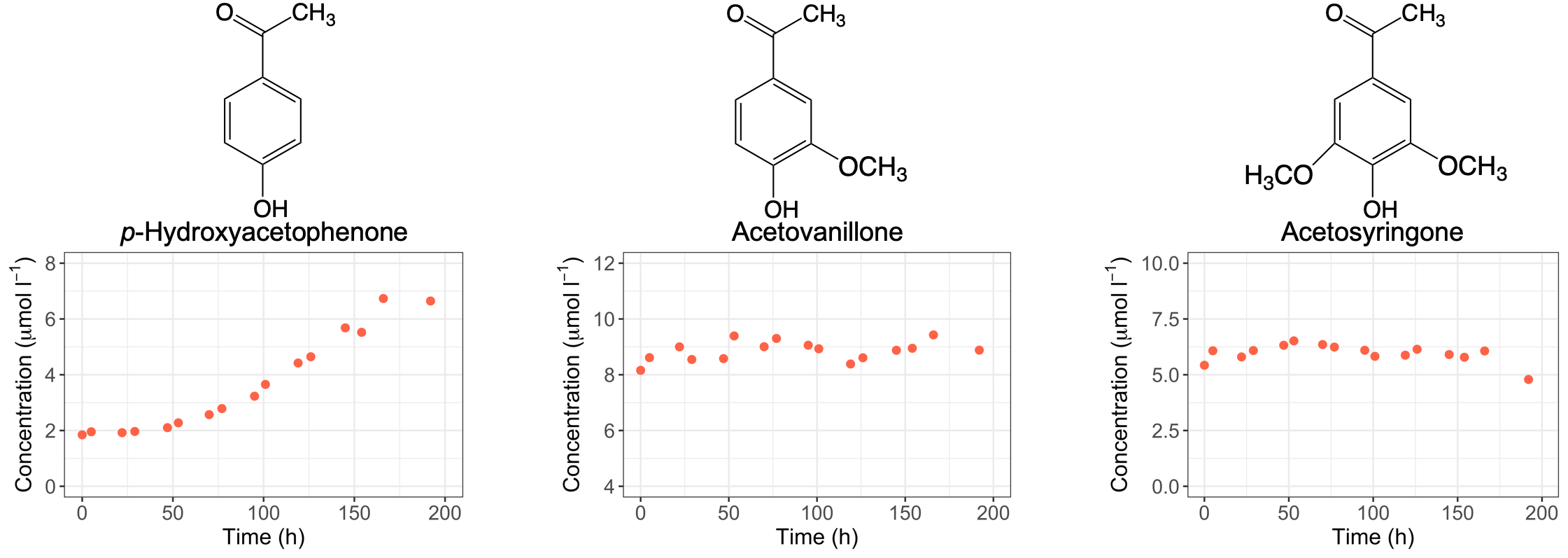
**

**Figure S3.** Kinetic modeling of (A) *p*-hydroxyphenyl, and (B) guaiacyl aromatics (black lines) compared to experimental results (red dots) corresponding to Case 1 in Fig. 2, Table 2 and 3. There are no inhibition effects or substrate channeling added in this model.

**
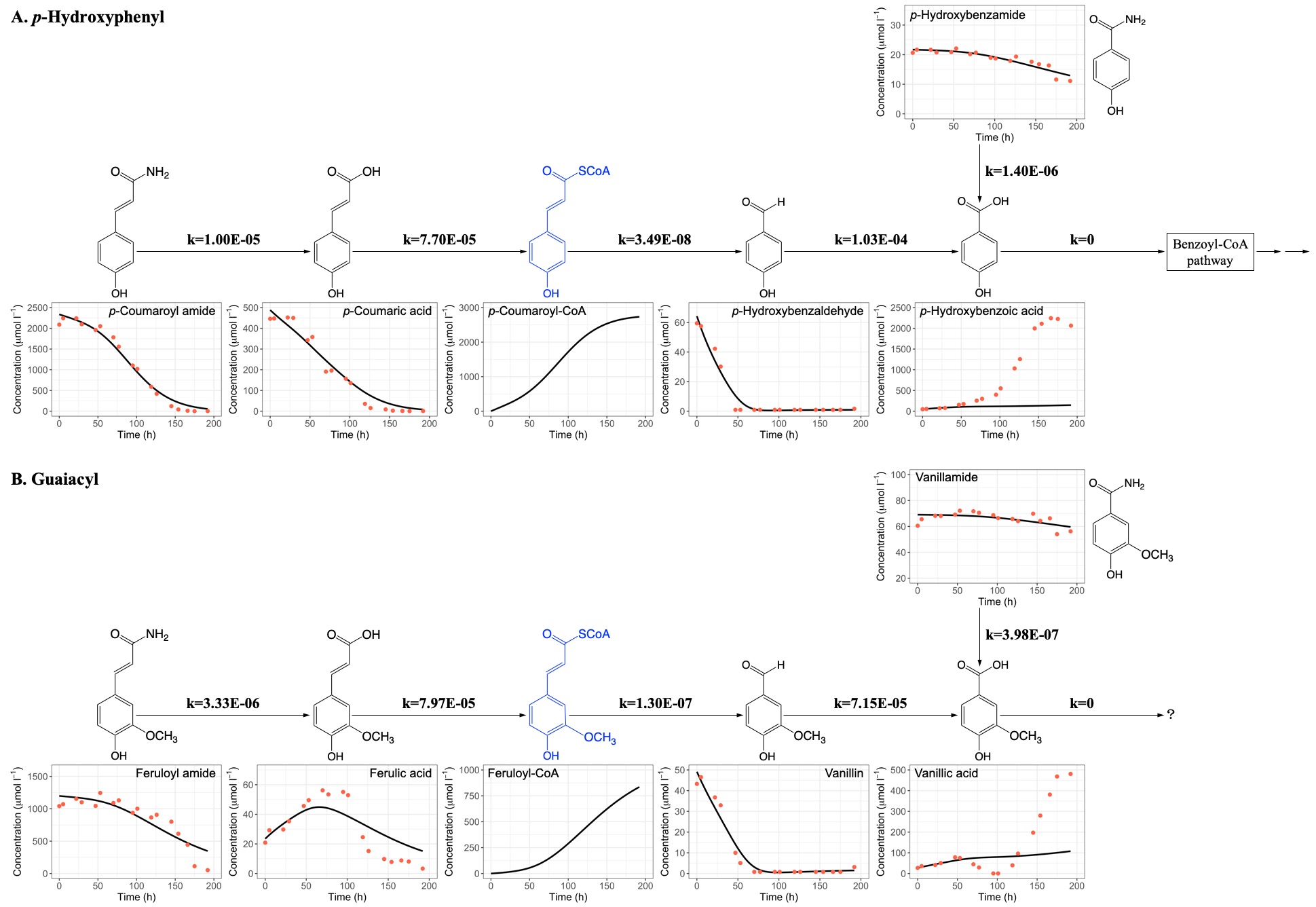
**

**Figure S4.** Kinetic modeling of (A) *p*-hydroxyphenyl, and (B) guaiacyl aromatics (black lines) compared to experimental results (red dots) corresponding to Case 2 in Fig, 2, Table 2 and 3. There are no inhibition effects added in this model. The dashed arrows between *p*-coumaroyl-CoA/feruloyl-CoA and *p*-hydroxybenzoic acid/vanillic acid represent substrate channeling, and thus *p*-hydroxybenzaldehyde and vanillin were not released to the bulk media.

**
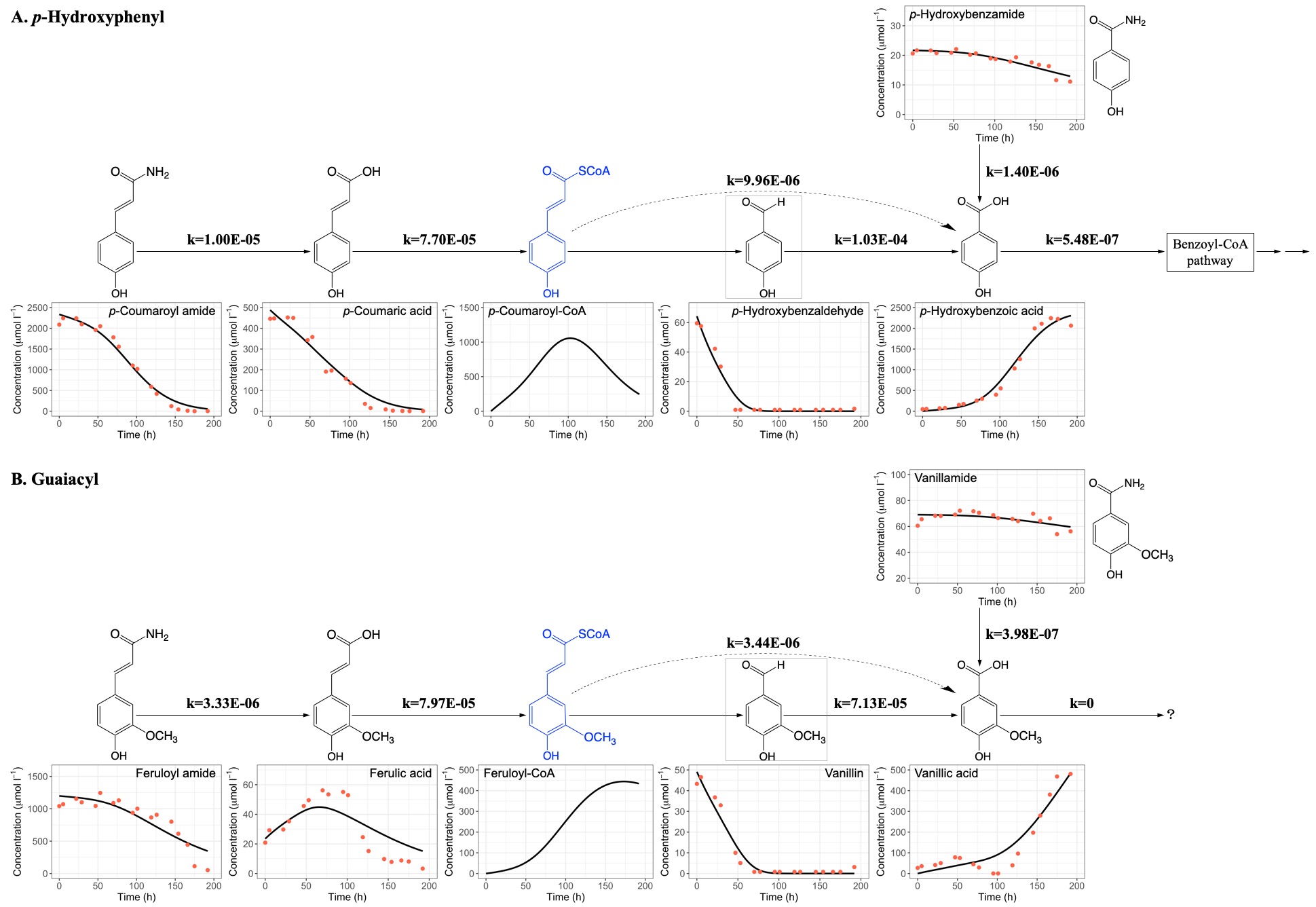
**

**Figure S5.** Kinetic modeling of (A) *p*-hydroxyphenyl, and (B) guaiacyl aromatics (black lines) compared to experimental results (red dots) corresponding to Case 3 in Fig. 2, Table 2 and 3. In this model, inhibition of H type aromatics to degradation of structurally similar G type aromatics was added. The dashed arrows between *p*-coumaroyl-CoA/feruloyl-CoA and *p*-hydroxybenzoic acid/vanillic acid represent substrate channeling, and thus *p*-hydroxybenzaldehyde and vanillin were not released to the bulk media.

**
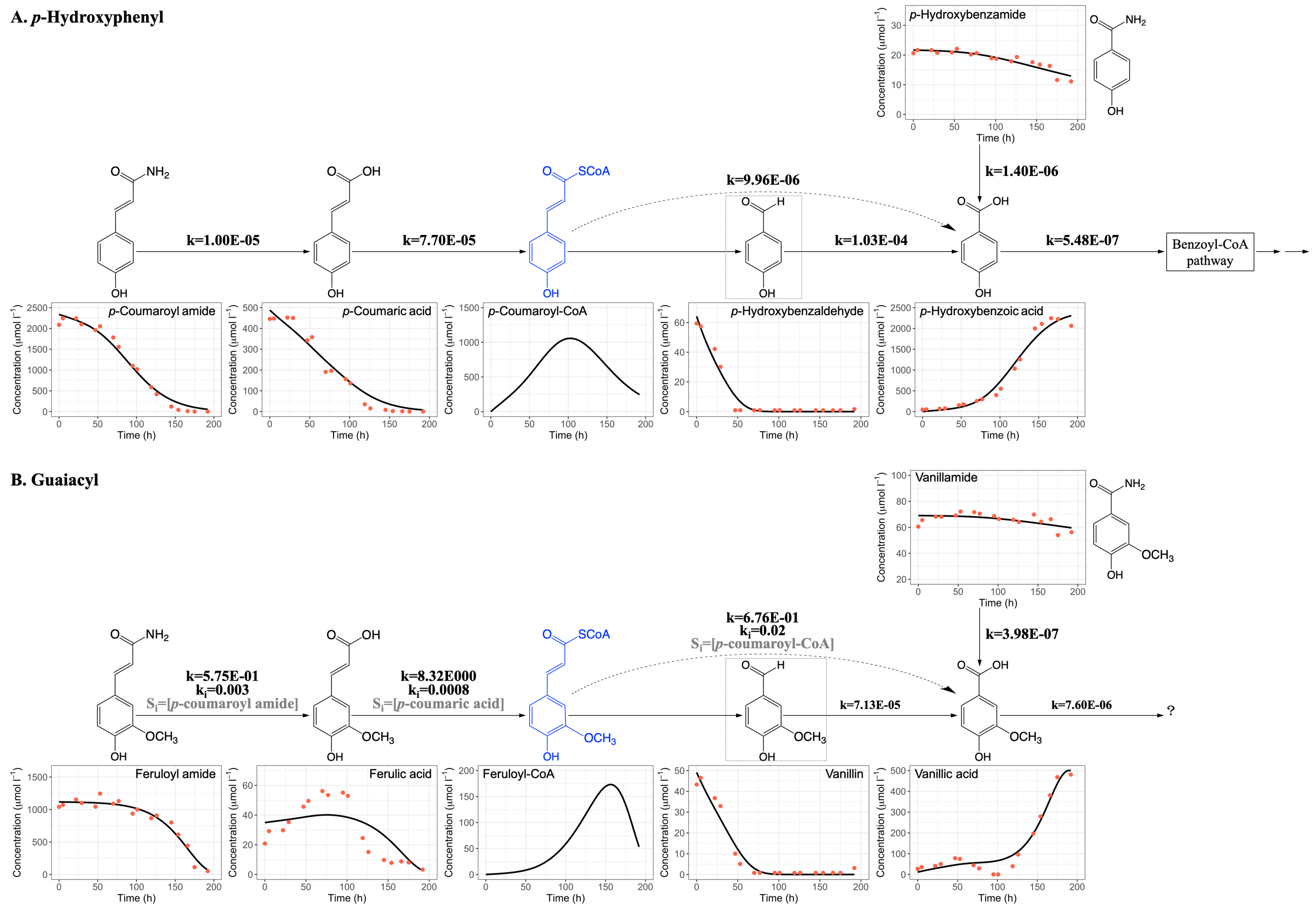
**

**Figure S6.** Kinetic modeling of (A) *p*-hydroxyphenyl, and (B) guaiacyl aromatics (black lines) compared to experimental results (red dots) corresponding to Case 4 in Fig. 2, Table 2 and 3. In this model, substrate inhibition was added to degradation of H and G type aromatics. The dashed arrows between *p*-coumaroyl-CoA/feruloyl-CoA and *p*-hydroxybenzoic acid/vanillic acid represent substrate channeling, and thus *p*-hydroxybenzaldehyde and vanillin were not released to the bulk media. “*” indicated the value of k_i_ is far larger than corresponding S_i_.

**
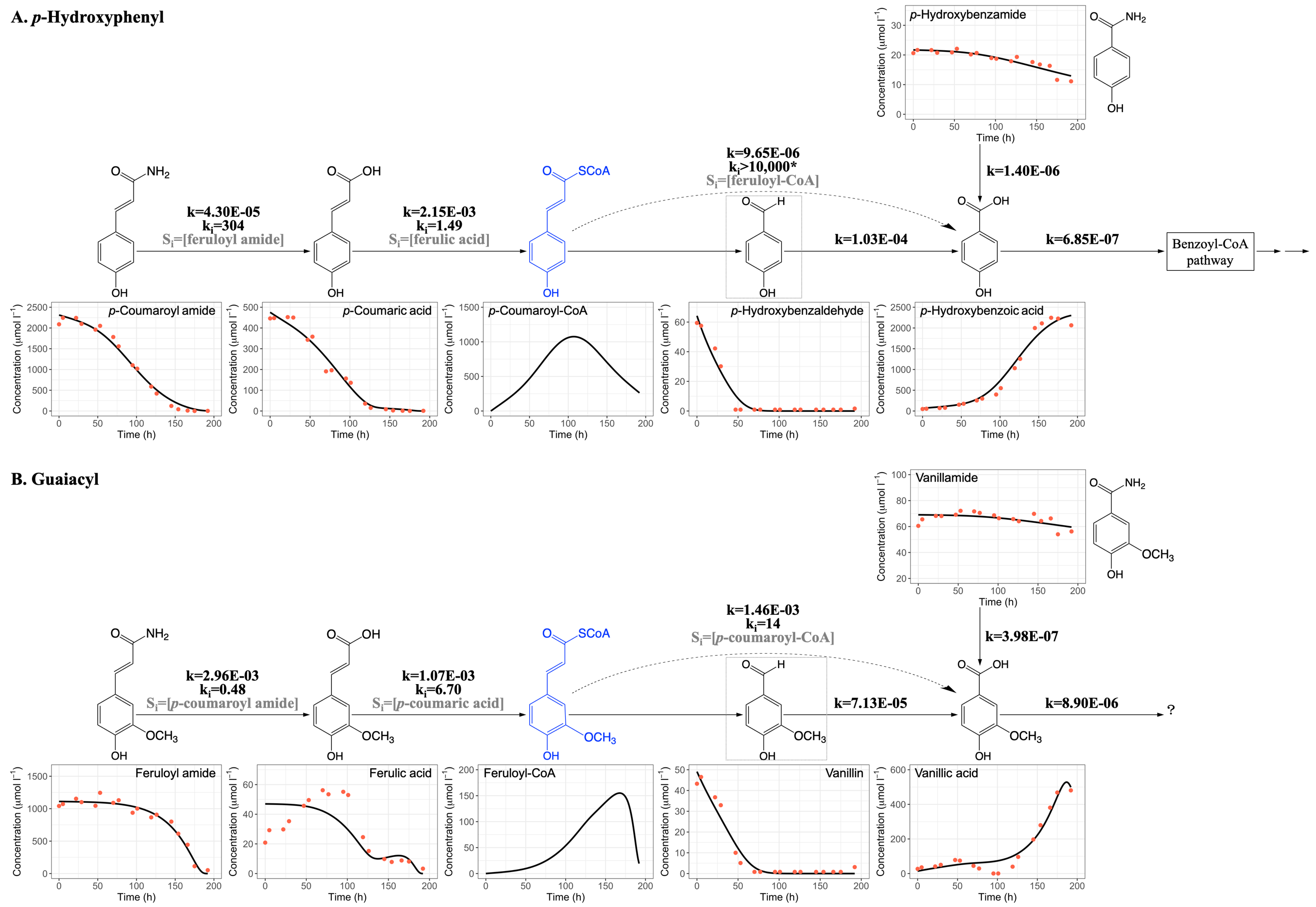
**

**Figure S7.** Kinetic modeling of (A) *p*-hydroxyphenyl, and (B) guaiacyl aromatics (black lines) compared to experimental results (red dots) corresponding to Case 5 in Fig2, Table 2 and 3. In this model, substrate inhibition was added to degradation of H type aromatics, and degradation of G type aromatics was assumed to be inhibited by structurally similar H type aromatics. The dashed arrows between *p*-coumaroyl-CoA/feruloyl-CoA and *p*-hydroxybenzoic acid/vanillic acid represent substrate channeling, and thus *p*-hydroxybenzaldehyde and vanillin were not released to the bulk media.


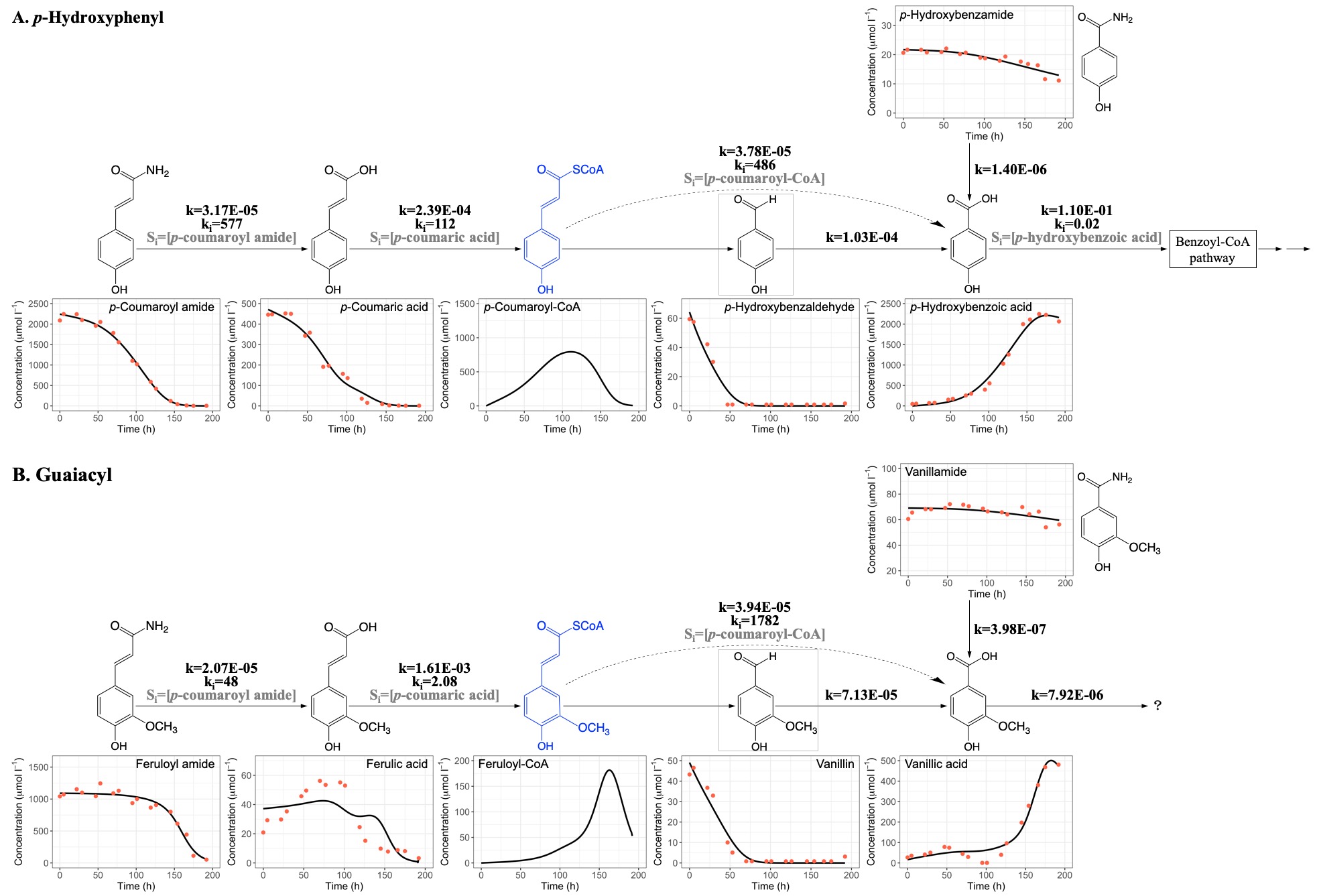


**Figure S8.** Modeling of biomass concentration (X) in the batch reactor using Gompertz function. Values of a, b and c were optimized by minimizing the Residual Sum of Squares (RSS) between the modeled and experimental results.


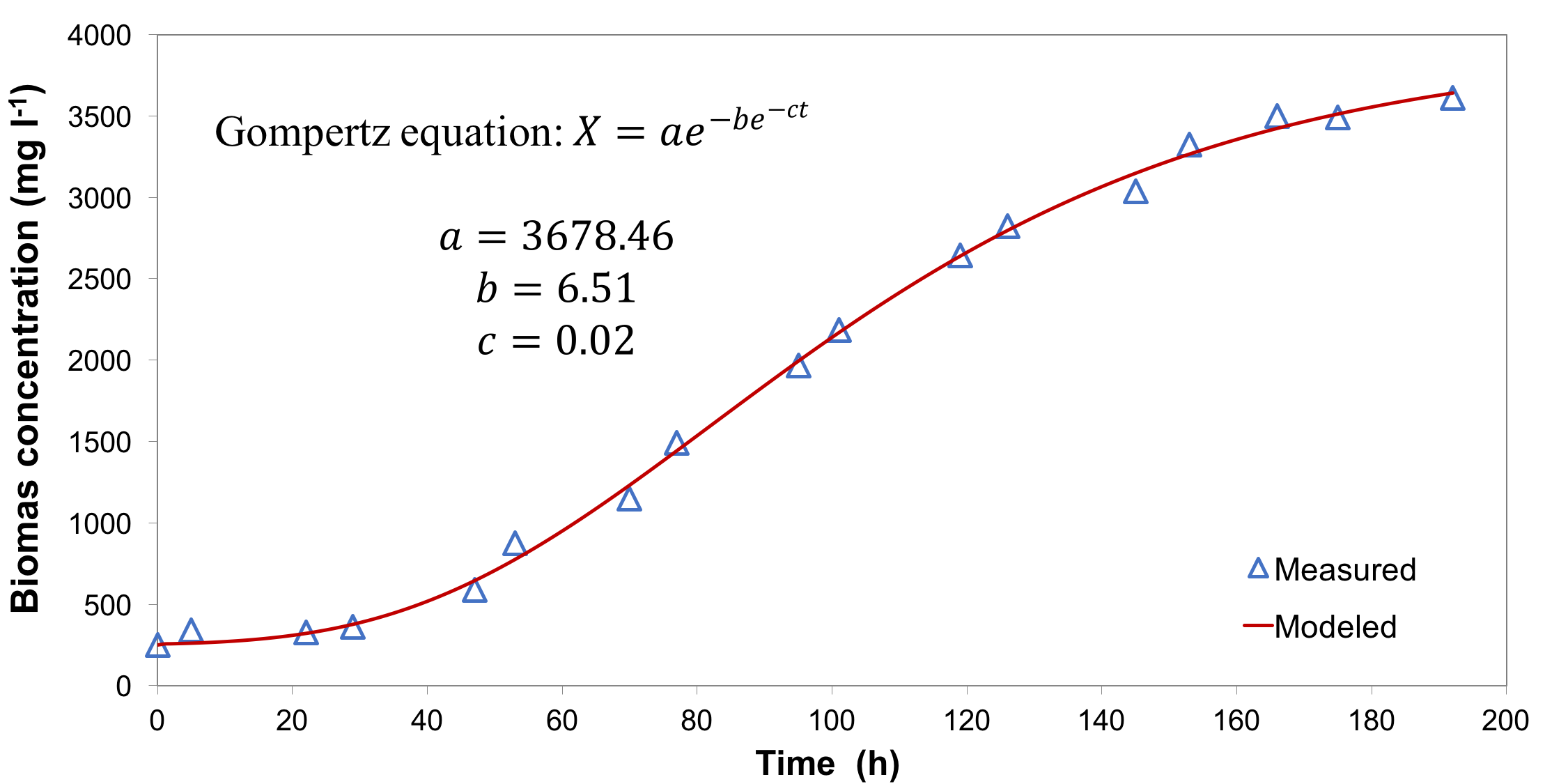


**References**

Austin, S., Kontur, W.S., Ulbrich, A., Oshlag, J.Z., Zhang, W.P., Higbee, A., Zhang, Y.P., Coon, J.J., Hodge, D.B., Donohue, T.J. and Noguera, D.R. (2015) Metabolism of Multiple Aromatic Compounds in Corn Stover Hydrolysate by *Rhodopseudomonas palustris*. *Environ Sci Technol* **49**, 8914-8922.
